# Modulation of surface phonon polaritons in MoO_3_ via dynamic doping of SiC substrate

**DOI:** 10.1515/nanoph-2024-0386

**Published:** 2024-12-06

**Authors:** Juan Luis Garcia-Pomar, Rajveer Fandan, Fernando Calle, Jorge Pedrós

**Affiliations:** Departamento de Ingeniería Electrónica, E.T.S.I. de Telecomunicación, Instituto de Sistemas Optoelectrónicos y Microtecnología (ISOM), Universidad Politécnica de Madrid, Madrid, 28040 Spain

**Keywords:** surface phonon polaritons, MoO_3_, SiC, doping, canalization, Purcell factor

## Abstract

Polar biaxial crystals with extreme anisotropy hold promise for the spatial control and the manipulation of polaritons, as they can undergo topological transitions. However, taking advantage of these unique properties for nanophotonic devices requires to find mechanisms to modulate dynamically the material response. Here, we present a study on the propagation of surface phonon polaritons (SPhPs) in a photonic architecture based on a thin layer of α-MoO_3_ deposited on a semiconducting 4H-SiC substrate, whose carrier density can be tuned through photoinduction. By employing this system, we establish a comprehensive polaritonic platform where the propagation of the hybridized SPhPs can be manipulated dynamically due to their coupling with the electron plasma. Specifically, we demonstrate that increasing the doping of the 4H-SiC substrate allows for modulating the on/off switch behavior of SPhP propagation or its controlled canalization. Furthermore, this modulation leads to a notable increase in the Purcell factor, primarily attributed to the doping-induced flat dispersion curve creating ultra-slow light. These findings have significant implications for the development of nanophotonic and quantum technologies, as they enable the utilization of polaritonic materials exclusively.

## 1 Introduction

Surface phonon polaritons (SPhPs) have recently emerged as a highly promising alternative to plasmonic counterparts, particularly in the mid-infrared (IR) to terahertz (THz) spectral range, for enhanced light–matter interactions and nanophotonic applications [1], [2], [3]. SPhPs are formed through the coupling of free-space photons with lattice vibrations in polar crystals, confined to sub-diffraction length scales near the surface with evanescent characteristics, and can support both localized and propagating surface modes resulting in extended lifetimes and minimal optical losses.

Molybdenum trioxide (α-MoO_3_), a 2D van der Waals biaxial crystal, has attracted a lot of attention as a standout polaritonic material offering configurable anisotropic phonon polaritons with high vertical confinement and long lifetime at frequencies ranging from the mid-IR to the far-IR [4]. In the three Reststrahlen bands of α-MoO_3_, the frequency ranges between transverse optical (TO) and longitudinal optical (LO) phonon frequencies, at least one component of the real part of the permittivity is negative, while the other two are positive, making the crystal hyperbolic, thus supporting hyperbolic phonon polaritons confined within these bands [5], [6], [7], [8]. In addition to in-plane hyperbolicity [9], the propagation of phonon polaritons in α-MoO_3_ can be tailored by means of twisted bilayers [8], [9], [10], [11], [12], [13], [14], [15], chemical intercalation [16], [17], or nanofabrication processes [9], [10], [18], [19], [20]. In particular, in the case of twisted α-MoO_3_ bilayers, phonon polaritons get canalized and thus propagate in one particular direction. The topology of the isofrequency dispersion contour of these polaritons can be controlled as a function of the twist angle, giving rise to exotic physics that provides avenues for novel polaritonic applications. However, a twisted heterostructure requires a very complicated fabrication process and lacks the capability for *in-situ* tuning, which is needed for developing tunable polaritonic devices. This latter limitation also applies to those approaches involving the chemical modification or physical patterning of α-MoO_3._ However, this restriction can be lifted in graphene/α-MoO_3_ heterostructures [14], [15], [21], [22], [23], [24], [25], [26] by means of the dynamic modulation of the graphene plasmons, that hybridize with the phonon polaritons in α-MoO_3_, provided by the voltage-induced doping of graphene [15], [25] (whereas chemically-doped graphene is just capable of providing a static modulation [19], [21], [22]). On the other hand, it has also been shown that the polaritonic dispersion can also be tailored selecting an appropriate substrate. For example, phonon polaritons in α-MoO_3_ hybridized with the phonon of an isotropic 4H-SiC substrate can be steered and excited unidirectionally [27], a capability not provided by a standard SiO_2_ substrate [16], [17].

**Figure 1:**
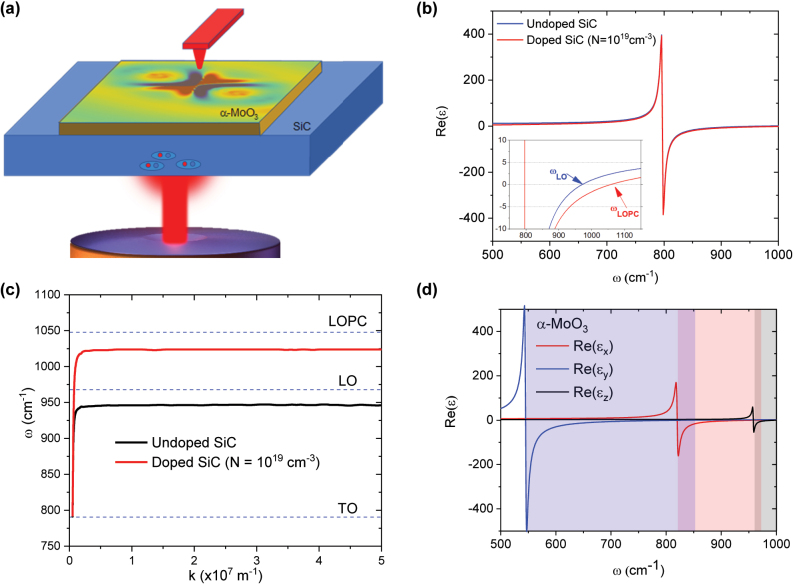
Photonic architecture and material dispersion analysis. (a) Schematic of the photonic architecture. (b) Real part of the permittivity of undoped and doped (*N* = 10^19^ cm^−3^) 4H-SiC. Inset shows the *ω*_LO_ and *ω*_LOPC_ frequencies for undoped and highly doped 4H-SiC. (c) Dispersion of undoped and doped (*N* = 10^19^ cm^−3^) 4H-SiC calculated by the transfer matrix method (TMM). The dashed lines indicate the TO and LO phonon frequencies of undoped 4H-SiC and the LOPC mode for doped 4H-SiC. *N* = 10^14^ cm^−3^ is considered the free carrier concentration (residual) in undoped 4H-SiC. (d) Real part of the three components of the permittivity of α-MoO_3_. Light blue, pink and gray zones correspond with the first, second, and third Reststrahlen bands, respectively.

In this article, we present theoretical research on the dynamic tunability of the propagation of SPhPs on a simple photonic architecture based on a thin layer of α-MoO_3_ on a 4H-SiC substrate (Figure 1(a)), offering significant advantages over previously reported methods by eliminating the need for complex or twisted heterostructure fabrication or intricate nanostructure engineering. The semiconducting nature of the 4H-SiC substrate not only enables the standard static doping through the introduction of impurities, but also dynamic doping through mechanisms such as voltage gating [28] or photoinduction [29], [30], which allows us to control the number of free carriers inside the material. Specifically, we demonstrate that photoinduction can tune the hybridized SPhP propagation in the α-MoO_3_/4H-SiC system by modulating the carrier density on the 4H-SiC substrate. In particular, we show that due to the large negative permittivity value of 4H-SiC, the hybridized SPhPs get canalized without the need of any external means and that the degree of canalization (or directionality) improves with increasing the 4H-SiC substrate doping, thus offering an alternative route towards realizing tunable canalized polaritons. We also show a full polaritonic band gap opening between 930 and 970 cm^−1^ when we introduce doping on the substrate, that provides a tunable on/off switch for the propagation of SPhPs in the α-MoO_3_/4H-SiC system, and we observe a remarkable enhancement of the Purcell factor at those frequencies for which the doping creates a flat dispersion curve. This flat-band condition involves slow light and high confinement phenomena which, as the enhancement in the Purcell factor indicates, strongly increases the light–matter interaction.

## 2 Results and discussion

The frequency-dependent permittivity of 4H-SiC is given by the permittivity at high frequency, *ɛ*_∞_ = 6.61, plus the Lorentz and the Drude terms, characterizing the behavior of the lattice and the free carriers, respectively [29], [30]:

(1) $$\varepsilon \left(\omega \right)=\varepsilon_{\infty }\left(1+\frac{\omega_{LO}^{2}-\omega_{TO}^{2}}{\omega_{TO}^{2}-\omega^{2}-i\omega \gamma }\right)-\underset{j=e,h}{\sum }\frac{\omega_{p\left(j\right)}^{2}}{\omega^{2}+i\omega \Gamma_{j}},$$

where *ω*_*LO*_, *ω*_*TO*_ and *γ* = 3.24 cm^−1^ are the LO, TO phonon frequencies and the damping rate, respectively, $\omega_{p\left(j\right)}$ is the plasma frequency, and Γ_*j*_ is the collision rate of the free carriers (*j* = *e*, *h* for electrons and holes, respectively). Both, $\omega_{p\left(j\right)}$ and Γ_*j*_ depend on the free-carrier density *N*_*j*_ as

(2) $$\omega_{p\left(j\right)}=\sqrt{\frac{N_{j}q^{2}}{\varepsilon_{0}m_{j}^{*}}},$$

(3) $$\Gamma_{j}=\frac{q}{m_{j}^{*}\mu_{j}\left(N_{j}\right)}.$$

Thus, $\omega_{p\left(j\right)}$ can be directly defined by *N*_*j*_, the electron charge *q*, the effective mass of the free carrier $m_{j}^{*}$ , and the vacuum permittivity *ɛ*_0_. Conversely, Γ_*j*_ depends indirectly on *N*_*j*_ through the carrier mobility *μ*_*j*_, as described by the Caughey–Thomas expression [31]

(4) $$\mu_{j}=\mu_{\min}+\frac{\mu_{\max}-\mu_{\min}}{1+{\left(\frac{N_{j}}{N_{0}}\right)}^{\alpha }},$$

that is used to fit the shape of the experimental curve of the mobility. In here, *μ*_min_ is the minimum mobility (at high *N*_*j*_), *μ*_max_ is the maximum mobility (at low *N*_*j*_), *N*_0_ is a characteristic carrier concentration, and *α* is a characteristic exponent. The empirical fitting parameters used are listed in Table S1 in the Supplementary Material, Section S1. At high carrier concentrations the plasma modes will couple to the LO lattice modes and will form the so-called LO–phonon–plasmon coupled (LOPC) modes [32], [33]. So, Equation (1) provides a way to understand how the presence of LOPC modes affects the permittivity of 4H-SiC, particularly in the frequency range where these modes are resonantly excited. This illustrates how the frequency-dependent permittivity of a polar semiconductor can be dynamically and significantly altered by introducing free carriers, providing active control of the optical and electronic properties in SPhP devices.

In this study, we have considered photoinduction doping where electron-hole pairs are generated in the 4H-SiC. It has been reported that a 355 nm pulsed laser with a pump fluence of 34 mJ/cm^2^ generates a carrier density of *N* = 10^19^ cm^−3^ [29]. It should be noted that, for other types of doping, the Drude term in Equation (1) would only consider either electrons or holes for *n*- or *p*-type doped 4H-SiC, respectively. For the sake of simplicity, we have assumed uniformly distributed carrier concentrations for both electrons, *N*_*e*_, and holes, *N*_*h*_ (see Supplementary Material Section S2). Furthermore, we have considered the injected carrier density to be significantly larger than the density of carrier traps, which has been reported to be of approximately 1 × 10^12^ cm^−3^ [34]. This ensured that the electron and hole carrier densities were approximately equal (*N* = *N*_*e*_ ≅ *N*_*h*_). We note that we have neglected the anisotropy of the dielectric response of 4H-SiC and considered an isotropic behavior, since *ε*_⊥_ and *ε*_||_ only differ by a small amount (see Supplementary Material Section S1). By making these assumptions, the analysis and calculations have been simplified, while they still maintain a reasonable approximation of the carrier densities in our model. Figure 1(b) shows the real part of the frequency-dependent permittivity of undoped 4H-SiC, that presents a Reststrahlen band in the mid-IR range, with TO and LO phonon frequencies at about 797 cm^−1^ (*λ* = 12.5 μm) and 970 cm^−1^ (*λ* = 10.3 μm), respectively [35], resulting in high reflectivity in that range. Figure S1 in the Supplementary Material Section S3 depicts a magnified view of the evolution of the real and imaginary parts of the 4H-SiC relative permittivity within the Reststrahlen band for increasing doping concentrations, that makes the real part of the permittivity values more negative. In addition, Figure 1(c) presents a scheme of the dispersion curves of undoped and doped 4H-SiC, where an 80 cm^−1^ blueshift of the LOPC mode appears relative to the LO phonon energy (970 cm^−1^) for undoped 4H-SiC. On the other hand, α-MoO_3_ has a frequency-dependent permittivity

(5) $$\varepsilon \left(\omega \right)=\left(\begin{array}{ccc} \varepsilon_{xx}\left(\omega \right) & 0 & 0\\ 0 & \varepsilon_{yy}\left(\omega \right) & 0\\ 0 & 0 & \varepsilon_{zz}\left(\omega \right) \end{array}\right),$$

where the trace terms, *ɛ*_*ii*_, are shown in Figure 1(d) for *i* = *x*, *y*, *z*. The rest of the parameters required for evaluating Equation (1) are listed in Section S4, Table S2 in the Supplementary Material. To complete the photonic overview of the material, Figure S2 in the Supplementary Material presents the calculated dispersion curve of an isolated self-standing thin film of α-MoO_3_.

In order to investigate the propagation of SPhPs in the α-MoO_3_/4H-SiC system, rigorous numerical simulations have been performed utilizing a finite-difference time-domain (FDTD) method. To excite the SPhPs, a vertical electric dipole source has been strategically positioned above the surface of the α-MoO_3_, as outlined in the Methods section. A 100-nm-thick layer of α-MoO_3_ deposited on a 4H-SiC substrate has been considered in order to obtain a hybridization between the SPhPs of α-MoO_3_ and of 4H-SiC, whose dielectric response can be tuned through the induced free carrier density in 4H-SiC. The study focuses in the Reststrahlen band of 4H-SiC, that spans from 790 to 970 cm^−1^.

### 2.1 Modulation of the SPhP dispersion

**Figure 2:**
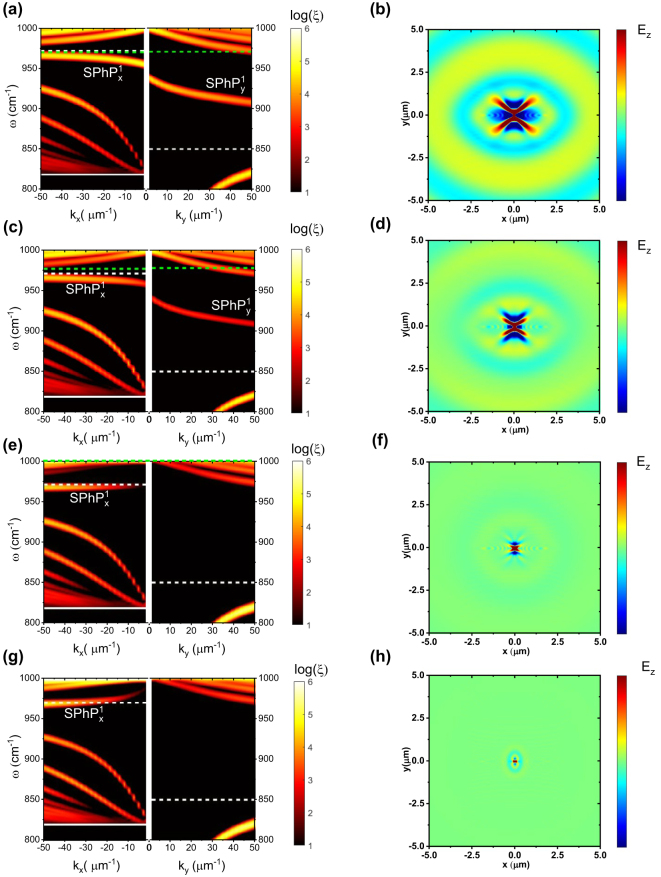
Dispersion curve extracted from the absolute value *ξ* of the spatial Fourier transform of the complex electric field (*E*_*Z*_) of the α-MoO_3_/4H-SiC system along the *k*_*x*_ and *k*_*y*_ directions for a 4H-SiC substrate with (a) no doping, (c) doping of *N* = 10^18^ cm^−3^, (e) *N* = 5 × 10^18^ cm^−3^, and (g) *N* = 10^19^ cm^−3^. The Reststrahlen band of α-MoO_3_ is indicated by the white solid (*ω*_TO_) and dashed (*ω*_LO_) lines, whereas the green dashed line indicates the *ω*_LO_ (*ω*_LOPC_) frequency for undoped (doped) 4H-SiC (*ω*_TO_ of 4H-SiC is out of the depicted ranges along the *k*_*y*_ direction and in (g) *ω*_LOPC_ is also out of the range). ${SPhP}_{x}^{1}$ and ${SPhP}_{y}^{1}$ are the modes discussed in detail in the main text. Real space *xy* mapping of the *z*-component of *E*_*z*_ at 940 cm^−1^ calculated 100 nm above the α-MoO_3_ surface for a 4H-SiC substrate with (b) no doping, (d) doping of *N* = 10^18^ cm^−3^, (f) *N* = 5 × 10^18^ cm^−3^, and (h) *N* = 10^19^ cm^−3^. *N* = 10^14^ cm^−3^ is considered the free carrier concentration (residual) in undoped 4H-SiC.

Figure 2 illustrates the evolution of the dispersion curves for the α-MoO_3_/4H-SiC heterostructure for different free carrier concentrations in the 4H-SiC substrate. The dispersion curves have been calculated by means of a FDTD method (see Methods) and verified by a transfer matrix method (TMM) (see Supplementary Material Section S5, Figures S3 and S4). We observe the creation of a polaritonic band gap in the frequency range of 930–960 cm^−1^ for doping concentrations higher than *N* = 5 × 10^18^ cm^−3^, as shown by the comparison of Figure 2(c) and (e). This provides the possibility of switching on/off the propagation of SPhPs by modifying the doping of the 4H-SiC substrate through photoinduction. The origin of this polaritonic band gap can be understood based on the fact that the Reststrahlen band of SiC broadens with the induced doping. At doping higher than *N* = 5 × 10^18^ cm^−3^, the Drude term in Equation (1) starts dominating over the Lorentz term. We have found that the frequency of the TO phonon of 4H-SiC remains unchanged with an increase in doping, while the LO phonon couples with carrier collective oscillations or plasmons, and this coupling modifies the 4H-SiC dielectric constant (Figure 1(b)). In this way, the macroscopic electric field associated with the plasmons interacts with the LO phonons by a deformation-potential + electro-optic (DP + EO) mechanism. This interaction modifies the Raman cross-sections and this leads to coupled modes of mixed phonon–plasmon character [36], [37]. With an increase in photoinduced carrier concentration in 4H-SiC, its LO phonon frequency shifts from 970 cm^−1^ (undoped) to 1,048 cm^−1^ (*N* = 1 × 10^19^ cm^−3^) and gets gradually damped due to the coupling with the photoinduced carriers forming the LOPC mode (see Figure S5 of the Supplementary Material). This LOPC mode hybridizes with the MoO_3_ modes, in both the *x*- and *y*-direction. Due to the anisotropic characteristic of MoO_3_ modes, their coupling with the LOPC mode is also anisotropic leading to hybridized SPhP modes with different spectral weight in *x*- and *y*-directions. In *y*-direction, the hybridized SPhP mode labelled as ${SPhP}_{y}^{1}$ in Figure 2, which spans from 900 to 950 cm^−1^ (as a function of *k*_*y*_) gets suppressed (i.e. vanishing character) with an increase in doping, as observed in Figure 2(e) and (g). Simultaneously, in the *x*-direction, the hybridized SPhP mode, labelled as ${SPhP}_{x}^{1}$ , reverses its curvature sign as a function of *k*_*x*_ with an increase in doping going through an intermediate flat band state at *N* = 5 × 10^18^ cm^−3^, as observed in Figure 2(e). Because of this concurrent coupling and modulation of the LOPC and the α-MoO_3_ SPhP modes in both *x*- and *y*-directions, a bandgap opens in the frequency range of 930–960 cm^−1^, as observed in Figure 2(e). The dynamic tuning and flat band achievement are the main highlights of this study as they lead to slow light and to an enhanced Purcell factor as discussed later. Note that this bandgap opening would have been identical if the slight anisotropic behavior of 4H-SiC would have been considered (see Figure S6 of the Supplementary Material).

Furthermore, Figure 2 shows the evolution of the propagation of SPhPs in real space at 940 cm^−1^ (which crosses the ${SPhP}_{y}^{1}$ branch for lowly doped 4H-SiC) for different photoinduced carrier concentrations, where clear propagation is observed for the undoped substrate (Figure 2(b)) while propagation is heavily suppressed for a doping level of *N* = 1 × 10^19^ cm^−3^ (Figure 2(h)) due to the bandgap opening in the frequency range of 930–960 cm^−1^ (Figure 2(g)). This paves the way for the dynamic modulation and tuning of the SPhPs in the α-MoO_3_/4H-SiC polaritonic structure. The same effect can also be observed in the corresponding isofrequency curves at 940 cm^−1^, Figure S7 in the Supplementary Material, showing also almost no coupling for *N* = 1 × 10^19^ cm^−3^ (Figure S7(d)).

### 2.2 Canalization of SPhPs

Within the overlapping frequency range of the Reststrahlen bands of the *x*-component of α-MoO_3_ and of 4H-SiC, we observe the phenomenon of canalization in the propagation of the polaritons at the α-MoO_3_ surface at 850 cm^−1^. Similar self-waveguiding or directional-beam phenomena have been demonstrated in various systems, such as electromagnetic waves in photonic crystals [38], [39], electrons in graphene [40], and, recently, phonon polaritons in twisted α-MoO_3_ [11], metal/α-MoO_3_/metal stacks [24], or patterned α-MoO_3_ [41]. The conditions for *x*-direction canalization in the system under consideration are: (1) the real part of the relative permittivity of the system in *y*-direction (orthogonal direction) must goes to zero and (2) the real part of the relative permittivity of the substrate (4H-SiC) must be negative. These conditions are fulfilled in the α-MoO_3_/4H-SiC system at 850 cm^−1^ and hence the canalization occurs (see the Supplementary Material Section S6, Figure S8). The canalization effect arises from the flat nature of the isofrequency curves and the perpendicular orientation of the group velocity *v*_*g*_, defined as $v_{g}=\nabla_{k}\omega \left(k\right)$ , with respect to these curves.

**Figure 3:**
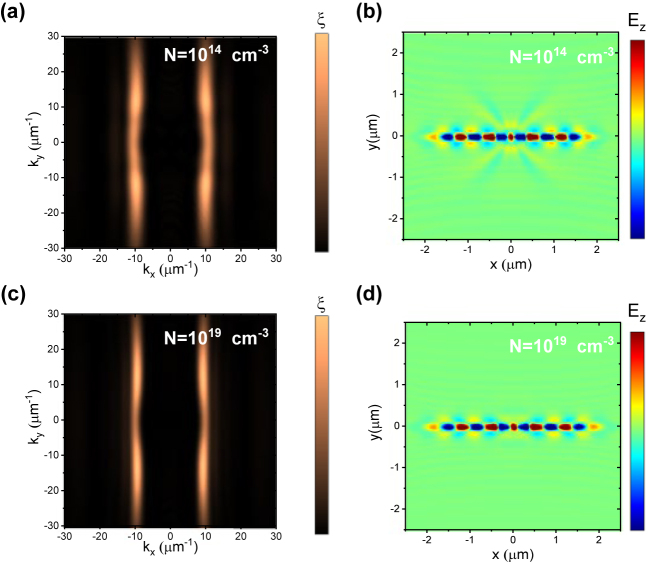
Isofrequency curves from the absolute value *ξ* of the spatial Fourier transform of the complex electric field (*E*_*Z*_) for the α-MoO_3_/4H-SiC system at 850 cm^−1^ for a 4H-SiC substrate with (a) no doping and (c) doping of *N* = 10^19^ cm^−3^ and their associated real space *xy* mappings of *E*_*z*_, calculated 100 nm above the α-MoO_3_ surface at the same frequency for a 4H-SiC substrate with (b) no doping and (d) doping of *N* = 10^19^ cm^−3^. *N* = 10^14^ cm^−3^ is considered the free carrier concentration (residual) in undoped 4H-SiC.

Figure 3(a) illustrates the calculated isofrequency curves at 850 cm^−1^ for the α-MoO_3_/4H-SiC system for undoped 4H-SiC calculated by means of FDTD (see Methods). The negative sign in the real part of the 4H-SiC permittivity at this frequency leads to flat isofrequency lines, indicating the presence of an image phonon polariton [42], [43] (see Figure S9 in the Supplementary Material) and a canalized beam on the α-MoO_3_ layer, as shown in Figure 3(b) in the real space mapping. These SPhPs have been described in systems based on ultrathin van der Waals materials on substrates with negative values of the real part of the permittivity [24], [27], [44]. By increasing the free carrier concentration to *N* = 1 × 10^19^ cm^−3^, the real part of the relative permittivity of 4H-SiC gets more negative and thus the isofrequency lines become even flatter (Figure 3(c)), resulting in a decrease in the dispersion angle *θ*_*d*_ of around 2°, as shown by the comparison of the real space images in Figure 3(b) and (d) for undoped and doped substrates, respectively.

### 2.3 Enhancement of the Purcell factor by SPhPs

**Figure 4:**
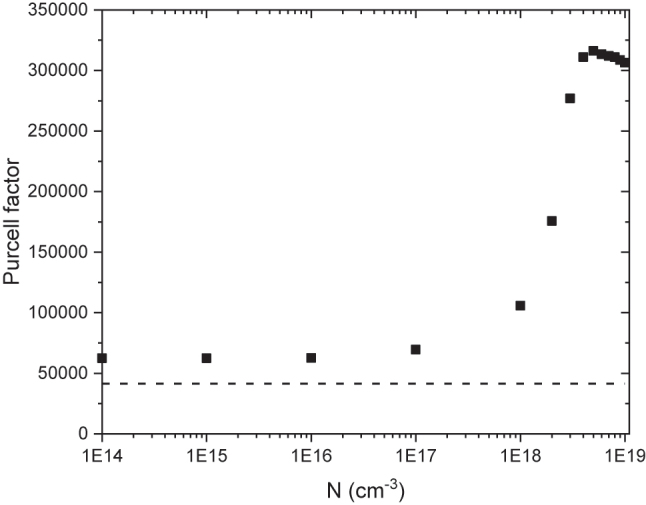
Purcell factor of the α-MoO_3_/4H-SiC system at 970 cm^−1^ for different free carrier concentration *N* values in the 4H-SiC substrate. The dashed line indicates the value of the Purcell factor for an α-MoO_3_ layer without substrate.

Furthermore, the SPhP dispersion in the α-MoO_3_/4H-SiC system indicates an ultra-slow group velocity for the *k*_*x*_ band around 970 cm^−1^ when the 4H-SiC substrate is doped with *N* = 5 × 10^18^ cm^−3^ (Figure 2(e)). The combination of slow light and high mode confinement has been shown to enhance significantly the Purcell factor of a quantum emitter placed above a polaritonic system [45]. We have calculated the Purcell factor of a quantum emitter located 10 nm above the α-MoO_3_/4H-SiC heterostructure at a frequency of 970 cm^−1^ (see Methods) for different doping concentrations of the 4H-SiC substrate, as plotted in Figure 4. It can be observed that the emission rate of a dipole placed within a 10 nm distance from the surface of the polaritonic structure can be made more than 5 times faster by tuning the free carrier concentration of the 4H-SiC substrate supporting the α-MoO_3_ layer. In Section S7 of the Supplementary Material, we have confirmed that this frequency of 970 cm^−1^ provides the maximum Purcell factor for a 4H-SiC substrate doped with *N* = 5 × 10^18^ cm^−3^ (Figure S10). The enhancement of the Purcell factor is expected to boost the performance of nanophotonic devices playing a crucial role in various applications, such as quantum optics and quantum communications. In particular, the mid-IR range and, more specifically, the range around 10 µm (∼970 cm^−1^), which corresponds to the optimum atmospheric transparency window [46], is of outstanding interest for free-space quantum key distribution (QKD) applications [47] or quantum interference phenomenon in biaxial crystals [26], [48], [49]. In this sense, the 10 nm distance above the α-MoO_3_ top surface considered here represents well the typical size of colloidal quantum dots [50], [51], that could be used as quantum emitters or single-photon sources in a QKD system operating in this optical range. Additionally, the change in the curvature of the dispersion curve for the *k*_*x*_ band around 970 cm^−1^, as shown by the comparison of Figure 2(c) and (e), besides providing a flat band condition leading to slow light, also leads to a change in the direction of the group velocity, opening new possibilities for tunable nano-polaritonic circuits [52]. Furthermore, it was found that the lifetime of polaritons on α-MoO_3_ appears to be less sensitive to substrate loss for negative values of the group velocity improving the SPhPs propagation [53].

## 3 Conclusions

Controlling the propagation of SPhPs, and thereby influencing the energy transport properties of subwavelength IR light fields, holds paramount importance for the effective utilization of nanophotonic devices. In this context, we propose a method to generate tunable hybridized SPhPs in α-MoO_3_ nanolayer based on the dynamic doping of the 4H-SiC substrate that holds it. We demonstrate an improvement in the SPhP canalization and the capability of switching on/off the SPhP propagation in the Reststrahlen band by varying the substrate doping via photoinduction or induced by voltage. This latter switching mechanism, related to an effective broadening of the Reststrahlen band of 4H-SiC, does not appear in the active modulation of SPhP modes of α-MoO_3_ by tuning the graphene Fermi energy (carriers), which only frequency shifts α-MoO_3_ SPhPs. In the case of the doped α-MoO_3_/4H-SiC system, 4H-SiC has both Lorentz (phonons) and Drude (carriers) terms (see Equation (1)). At low doping, 4H-SiC acts as a dielectric with a dominant Lorentz term that leads to a Reststrahlen band. However, by increasing the doping, the Drude and Lorentz terms interplay leading to the emergence of the LOPC mode that effectively broadens the 4H-SiC Reststrahlen band, and the opening of a polaritonic bandgap in the α-MoO_3_/4H-SiC system.

In general, similar results are expected to be obtained with any combination of a hyperbolic material with a semiconductor substrate with the following requirements: (i) the semiconductor should facilitate a tunable free carrier density, which in turn leads to the modulation/coupling with its LO phonon, forming a LOPC mode and, hence, to the broadening of its Reststrahlen band; (ii) there should be an overlap between the Reststrahlen bands of the hyperbolic and the semiconductor materials; (iii) the LOPC mode, created at high doping levels, should be at a higher frequency than the LO phonon in both in-plane directions (*x* and *y*) of the hyperbolic material. For example, V_2_O_5_, a hyperbolic material similar to α-MoO_3_, could provide similar results, such as canalization and controllable bandgap opening when placed on top of 4H-SiC (or other polytypes of SiC).

Finally, using this tunability, we can obtain a slow light mode that increases the Purcell factor above the α-MoO_3_ nanostructure, offering new perspectives for enhanced light–matter interaction and quantum optics. Therefore, the insight provided not only offers essential understanding into the precise control of light through polaritonic crystals at the nanoscale, but also unveils novel possibilities for tunable nanophotonic applications. Hence, understanding the impact of the carrier concentration on the propagation behavior of hybridized SPhPs is crucial for achieving dynamic tunability in polaritonic systems and serves as a foundation for developing several applications, including tunable optical waveguides and resonators, as well as enhanced single-photon sources.

## 4 Methods

Simulations have been conducted using the Ansys Lumerical [54] FDTD solver for Maxwell’s equations to analyze the electric field distributions on α-MoO_3_/4H-SiC heterostructures. The simulations were carried out considering the interaction with a point dipole source, positioned 100 nm above the top surface of the α-MoO_3_/4H-SiC structure. The electric dipole was polarized along the *z* direction, and electric field monitors were strategically placed on the top surface for data collection. To ensure accurate results, a perfectly matched layer (PML) was employed for the boundary conditions. The field signal was apodised below 400 fs to exclude the dipole source excitation from the frequency-domain data.

The SPhP band structure of the α-MoO_3_/4H-SiC heterostructure, considering different 4H-SiC doping concentrations, was computed using the FDTD method provided by Lumerical. A residual free carrier (doping) concentration of *N* = 1 × 10^14^ cm^−3^ has been considered for the undoped case. To obtain the dispersion curves, a small number of TE-polarized point dipoles were randomly positioned within a unit cell that contains a nanoparticle. This configuration allows for the excitation of TE-like modes in the heterostructure. Bloch boundary conditions were applied along the *x* and *y* axes, while PML conditions were used along the *z* axis. Additionally, a set of 10 time-monitors was randomly distributed within the unit cell to measure the electric field. The recorded electric field data was then post-processed using Fourier transform to identify resonances in the frequency domain. By repeating this process for different *k* vectors in the Bloch boundary conditions, the complete SPhP band structure of the heterostructure was obtained. The validity of this method has been verified by means of the transfer matrix method (TMM) [55], [56], [57], as shown in the Supplementary Material, Section S5. The calculation of the Purcell factors was also conducted utilizing Lumerical. The Purcell factor is directly derived from the outcome of the source, which represents a vertically-oriented electrical dipole situated 10 nm above the α-MoO_3_ top surface, a distance that represents the typical size of a quantum emitter [47], [48].

## Supplementary Material

Supplementary Material Details
