# Supplementary material for: Modulation of surface phonon polaritons in MoO3 via dynamic doping of SiC substrate
Source: Nanophotonics. 2024 Dec 6;14(1):23–32. doi: 10.1515/nanoph-2024-0386 (PMC11744456; doi:10.1515/nanoph-2024-0386)
Supplement: Supplementary file 1 — Supplementary Material Details [file j_nanoph-2024-0386_suppl_001.pdf]

Supplementary Material

Modulation of Surface Phonon Polaritons in MoO_3 via dynamic doping of SiC substrate

Juan Luis Garcia-Pomar, Rajveer Fandan, Fernando Calle, and Jorge Pedrós**

Instituto de Sistemas Optoelectrónicos y Microtecnología (ISOM) and Departamento de Ingeniería Electrónica, E.T.S.I. de Telecomunicación, Universidad Politécnica de Madrid, Madrid, 28040 Spain

* E-mail: jl.garcia.pomar@upm.es, j.pedros@upm.es

Contents:

S1. Parameters for 4H-SiC

S2. Approximations in the photoinduced carrier density in 4H-SiC

S3. Evolution of the permittivity with the doping in 4H-SiC

S4. Parameters for α-MoO_3

S5. Dispersion curves of the α-MoO_3/4H-SiC system calculated by the transfer matrix method

S6. Conditions for canalization and effects of doping on it

S7. Purcell factor calculated for a 4H-SiC substrate doped with $N = 5 \times 10^{18}$ cm^{-3}

S1. Parameters for 4H-SiC

The parameters of the electron and hole mobility models and effective masses are summarized in Table S1. For the effective mass of the electrons and holes we used the transverse component from Refs. [1], [2] , respectively. The mobility at room temperature has been taken from Refs. [3] for the electrons and holes, respectively.

Table S1. Experimental fitting parameters for the 4H-SiC

Parameter [units]	μ_{max} [cm^2/Vs]	μ_{min} [cm^2/Vs]	N_0 [$\times 10^{17}$ cm^{-3}]	α	m^*/m_e
Electron	950	40	2	0.76	0.44
Hole	120	15.9	18	0.65	0.66

These parameters, together with ε_∞, ω_{TO} and ω_{LO} given by Ref. [4], allows us to obtain the frequency-dependent permittivity of 4H-SiC for different values of the free carrier density (see Figure 1(c) of the main text). We note that the mobility given by the photoinduced free carriers has an empirical fitted factor of the mobility of the impurities [5], which we have considered to be close to 1. This assumption has allowed us to calculate the dependence of the permittivity with the carrier concentration, providing values that fit quite well with those obtained by Ref. [6]. It should be noted as well that an isotropic approximation has been used for the permittivity of 4H-SiC, which relies on the excellent agreement reported in the literature using this approximation for the description of SPhP modes [7-10]. This approximation has also been successfully used for describing shallow donors [11] and photoinduced carriers [6].

S2. Approximations in the photoinduced carrier density

The penetration depth of a pulsed laser in 4H-SiC has been reported to be approximately of 50 μm and a stationary profile of photoinduced carriers is reached in around 2 μs [12]. Therefore, a uniform carrier distribution can be assumed in a 4H-SiC substrate of a thickness ≤ 50 μm while it is being continuously pumped by the laser. On the other hand, SPhPs penetrate just around 500 nm into the substrate in a α-MoO_3/4H-SiC system, as shown in Figure S9. Experimentally, photoinduction can be produced in 4H-SiC thin films, grown epitaxially on AlN/sapphire substrates (which are transparent to the laser excitation of photocarriers) [13-16] It is worth noting that while our manuscript primarily focuses on 4H-SiC, the same phenomenon would apply to other polytypes of SiC.

S3. Evolution of the permittivity with the doping in 4H-SiC.

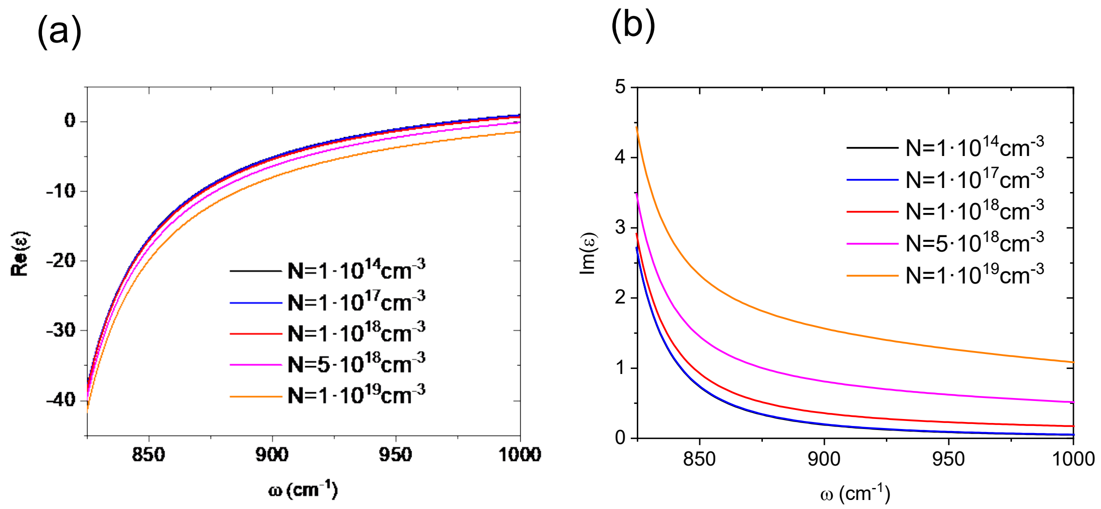

Figure S1. *Tunability of the (a) real and (b) imaginary parts of the permittivity of 4H-SiC in the reststrahlen band by doping. N = 10^{14} cm^{-3} is considered the free carrier concentration (residual) in undoped 4H-SiC.*

S4. Parameters for α-MoO_3

The optical parameters for the calculation of the frequency-dependent permittivity of α-MoO_3 are given in Table S2.[17]

Table S2. Parameters for α-MoO_3

Parameter [unit]	ε_∞	ω_{TO} [cm^{-1}]	ω_{LO} [cm^{-1}]	γ [$\times 10^{11}$ rad/s]
xx	4	820	972	7.5345
yy	5.2	545	851	7.5345
zz	2.4	958	1004	3.7673

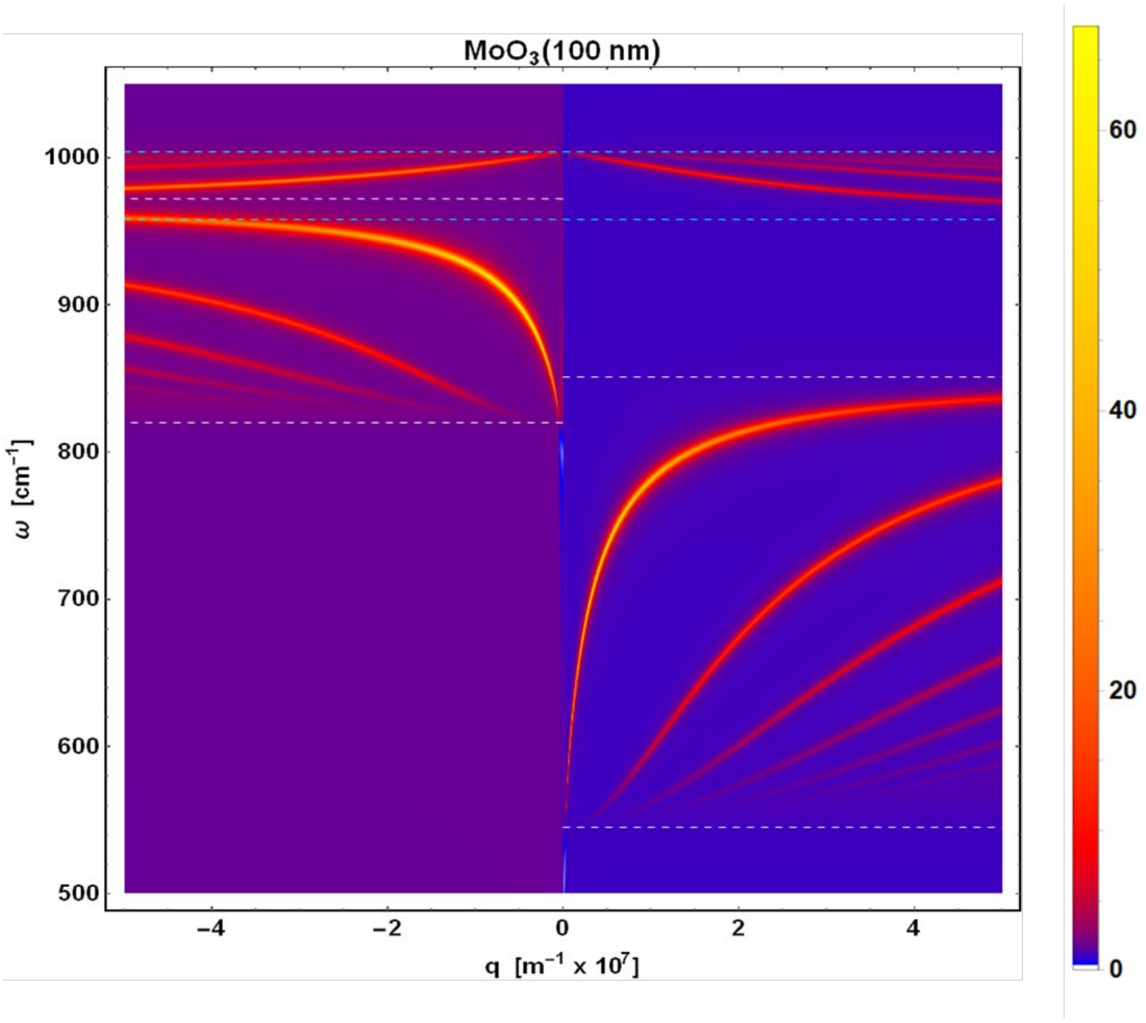

Figure S2. *Dispersion of a self-standing thin film of α-MoO_3 calculated by the transfer matrix method (TMM). Left (negative) and right (positive) sides of the abscissa axis represent q_x and q_y wavevectors, respectively. White dashed lines represent the TO (bottom) and LO (top) phonon frequencies of α-MoO_3 in the x and y directions, whereas the blue lines correspond to the TO (bottom) and LO (top) phonon frequencies along the z direction. The scale bar represents the imaginary part of the p-polarized Fresnel reflection coefficient.*

S5. Dispersion and isofrequency curves of the α-MoO_3/4H-SiC system calculated by the transfer matrix method, comparison for anisotropic 4H-SiC.

The dispersion curves calculated by FDTD method were verified by calculation via the transfer matrix method (TMM) [18].

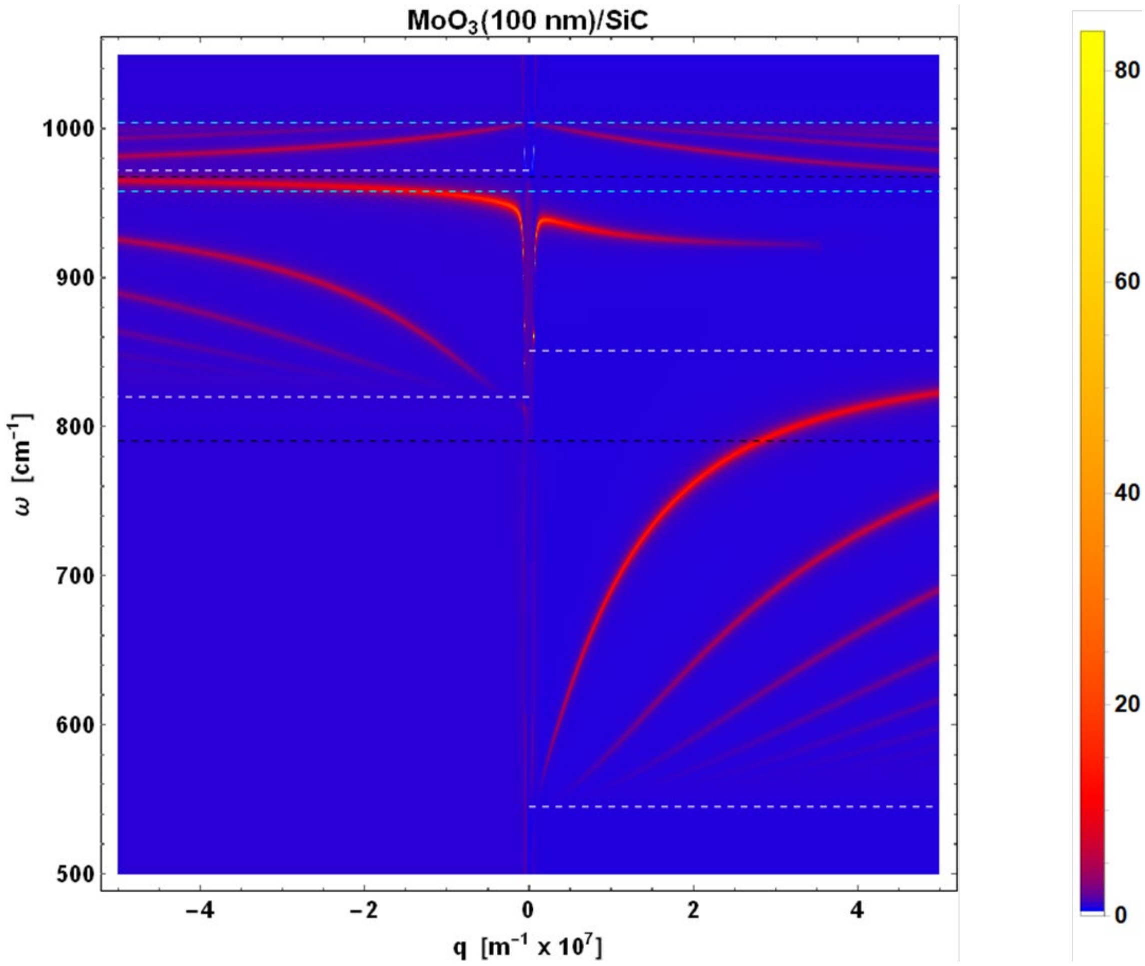

Figure S3 *Dispersion of an α-MoO_3/4H-SiC system, where the 4H-SiC substrate has no doping, calculated using the transfer matrix method (TMM). Left (negative) and right (positive) sides of the abscissa axis represent q_x and q_y wavevectors, respectively. White dashed lines represent the TO (bottom) and LO (top) phonon frequencies of α-MoO_3 in the x and y directions, whereas the blue lines correspond to the TO (bottom) and LO (top) phonon frequencies along the z direction, and the black lines indicate the TO (bottom) and LO (top) phonon frequencies of 4H-SiC. The scale bar represents the imaginary part of the p-polarized Fresnel reflection coefficient.*

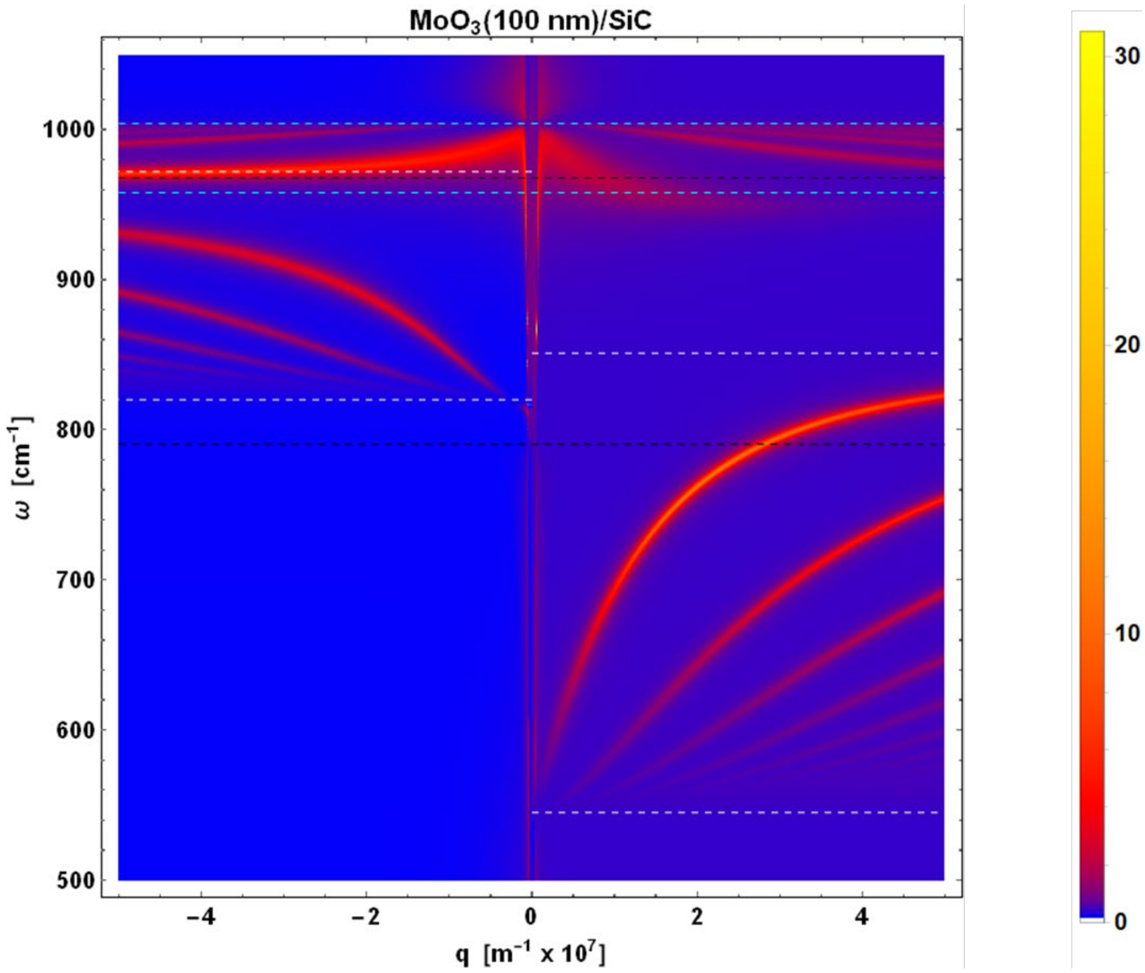

Figure S4. *Dispersion of an α-MoO_3/SiC system, where the 4H-SiC substrate doping is N = 10^{19} cm^{-3}, calculated using the transfer matrix method (TMM). Left (negative) and right (positive) sides of the abscissa axis represent q_x and q_y wavevectors, respectively. White dashed lines represent the TO (bottom) and LO (top) phonon frequencies of α-MoO_3 in the x and y directions, whereas the blue lines correspond to the TO (bottom) and LO (top) phonon frequencies along the z direction, and the black lines indicate the TO (bottom) and LO (top) phonon frequencies of 4H-SiC. The scale bar represents the imaginary part of the p-polarized Fresnel reflection coefficient.*

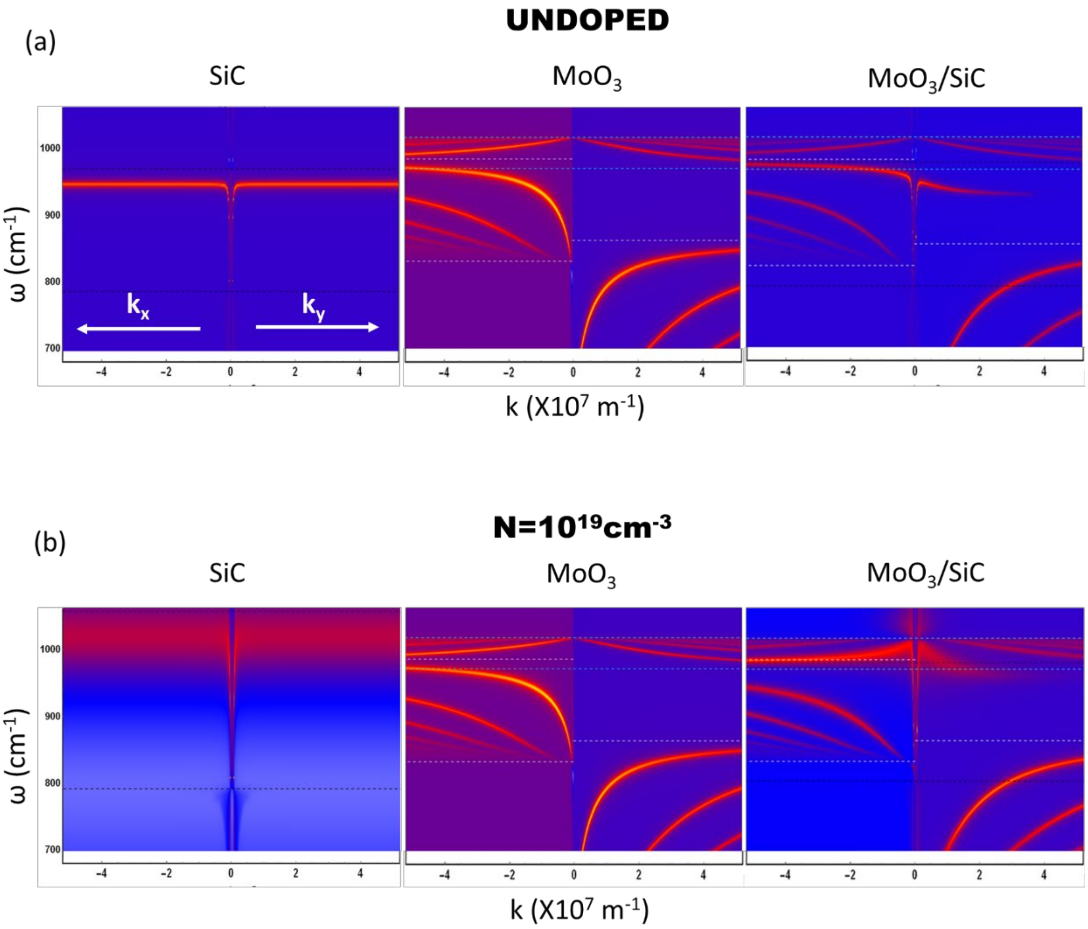

Figure S5. *Scheme of the hybridization process for low and high doping levels. Dispersion of 4H-SiC, α-MoO_3 (100 nm thick) and α-MoO_3/4H-SiC system calculated using the transfer matrix method (TMM), where the 4H-SiC is (a) undoped (N = 10^{14} cm^{-3}) and (b) with a doping of N = 10^{19} cm^{-3}. Left panel (4H-SiC) shows the SPhP of 4H-SiC and the formation of the LOPC for a doping level of N = 10^{19} cm^{-3}. Middle panel (α-MoO_3) shows the phonon polaritons in the reststrahlen bands. Right panel (α-MoO_3/4H-SiC) shows the hybridized modes of the whole system for the undoped and doped cases.*

Figure S6 presents the comparison of the dispersion curves of an α-MoO_3/4H-SiC system considering the isotropic approximation (left panel) and the full anisotropic (right panel) permittivity of the 4H-SiC substrate for undoped (N = 10^{14} cm^{-3}) and doped (N = 10^{19} cm^{-3}), conditions. It can be noted that the same bandgap opening is observed in both cases, with just a slightly shift of the polaritonic bandgap to higher frequencies in the anisotropic case.

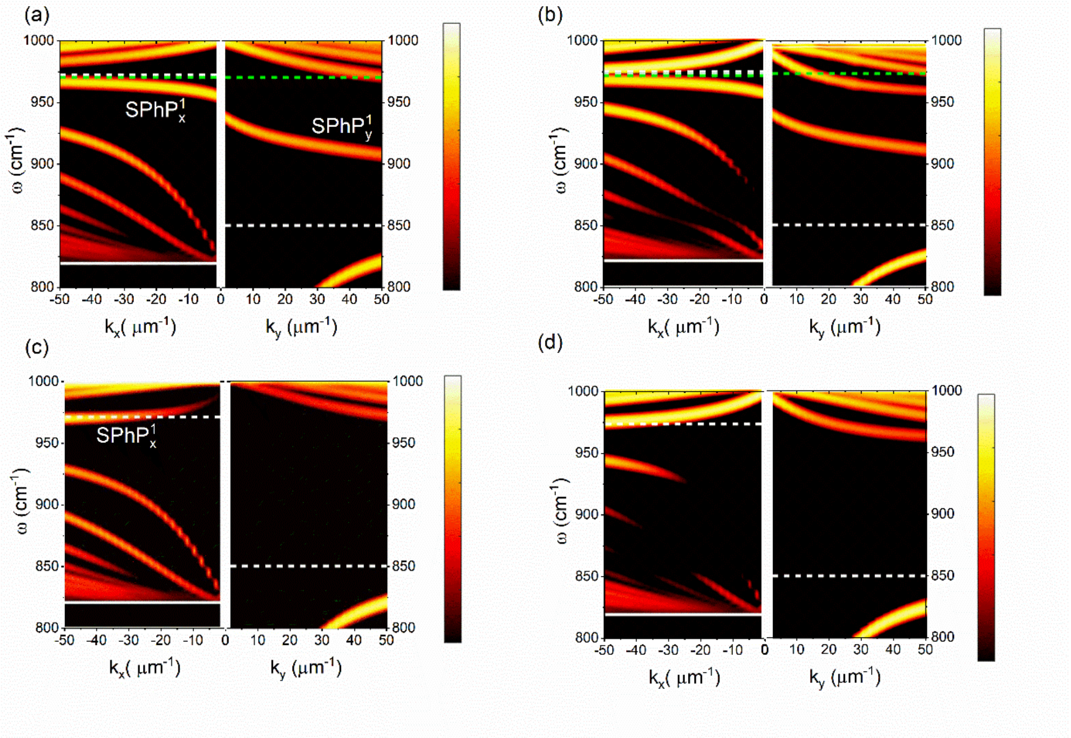

Figure S6. *Dispersion of the α-MoO_3/4H-SiC system along the k_x and k_y directions for a 4H-SiC substrate for the isotropic approximation with (a) no doping, (c) doping of N = 10^{19} cm^{-3}, and for the anisotropic calculation with (b) no doping and (d) doping of N = 10^{19} cm^{-3}. The reststrahlen band of α-MoO_3 is indicated by the white solid (ω_{TO}) and dashed (ω_{LO}) lines, whereas the green dashed line indicates the ω_{LO} (ω_{LOPC}) frequency for undoped (doped) 4H-SiC (ω_{TO} of 4H-SiC is out of the depicted ranges along the k_y direction and in (c) and (d) ω_{LOPC} is also out of the range). Material parameter used in the anisotropic case: ω_{LO}^z (SiC) = 964 cm^{-1}, ω_{TO}^z (SiC) = 782 cm^{-1}, γ^z (SiC) = 3.54 cm^{-1}, and ε_∞^z (SiC) = 7.32. The scale bar represents the absolute value of the spatial Fourier transform of the complex electric field (E_Z).*

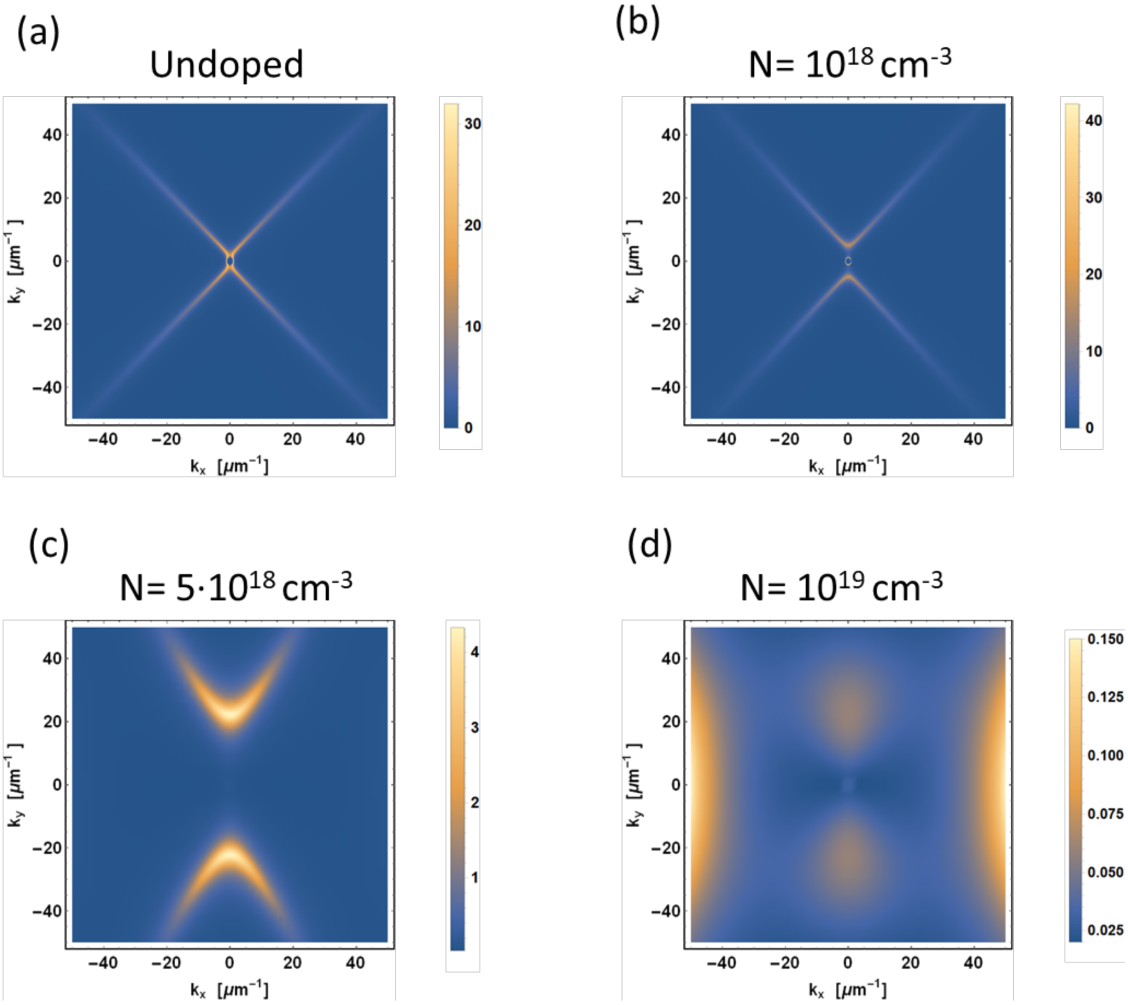

Figure S7. *Isofrequency curves of α-MoO_3/SiC system, where the 4H-SiC substrate doping is (a) undoped, (b) N = 10^{18} cm^{-3}, (c) N = 5 × 10^{18} cm^{-3} and (d) N = 10^{19} cm^{-3}, at 940 cm^{-1}. The scale bar represents the imaginary part of the p-polarized Fresnel reflection coefficient. (d) For a doping of N = 10^{19} cm^{-3} the coupling is almost null in comparison with the scales of the lower doping levels.*

S6. Conditions for canalization and effects of doping on it

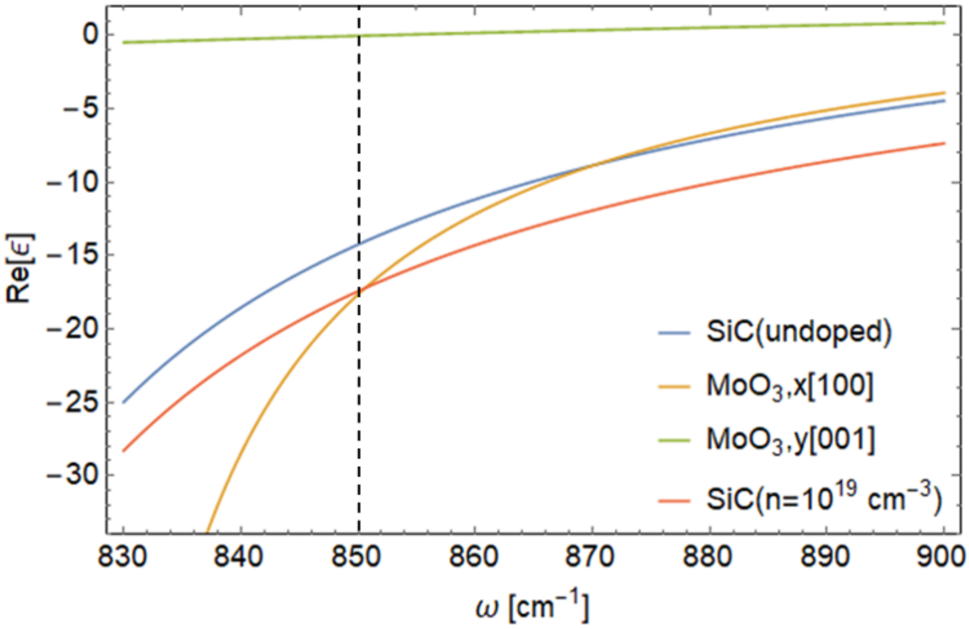

Figure S8 *Conditions for canalization and the effects of doping on it. For canalization in the x-direction at 850 cm^{-1}, the permittivity of α-MoO_3 in the x-direction is negative, the permittivity of α-MoO_3 in the y-direction is zero, and the permittivity of undoped 4H-SiC is negative. With an increase in doping, the permittivity of 4H-SiC gets more negative.*

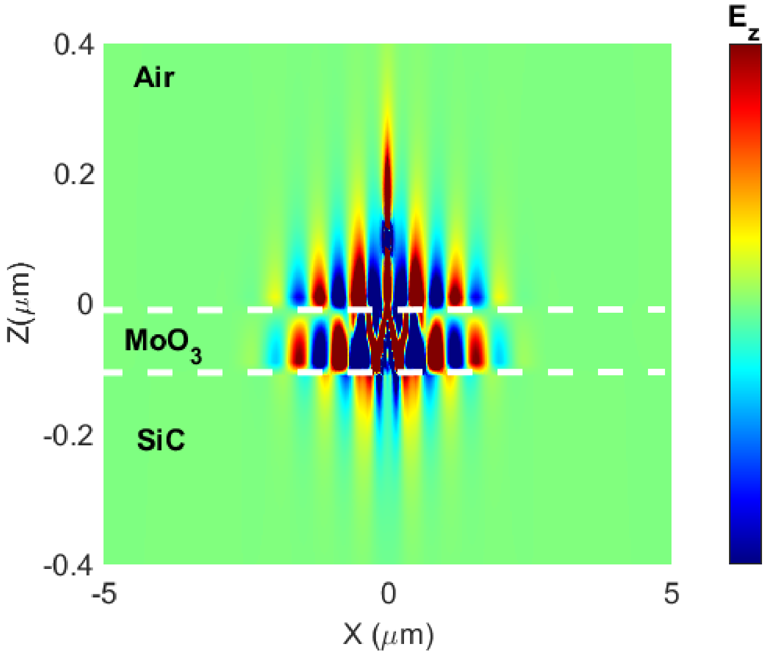

Figure S9. *Transversal cut of a FDTD calculation at 850 cm^{-1} of the z-component of the electric field for the XZ plane, for an emitter situated 100 nm above an α-MoO_3/4H-SiC system with a substrate with a carrier concentration of N = 10^{19} cm^{-3} showing an image polariton* [19].

S7. Purcell factor calculated for a 4H-SiC substrate doped with $N = 5 \times 10^{18}$ cm^{-3}

The Purcell factor results from dividing the power emitted by a dipole source placed 10 nm above the α-MoO_3/4H-SiC system by the power emitted by the same dipole source alone, since the spontaneous emission rate is proportional to the local density of states (LDOS), and the latter is proportional to the power emitted by the source [20, 21]

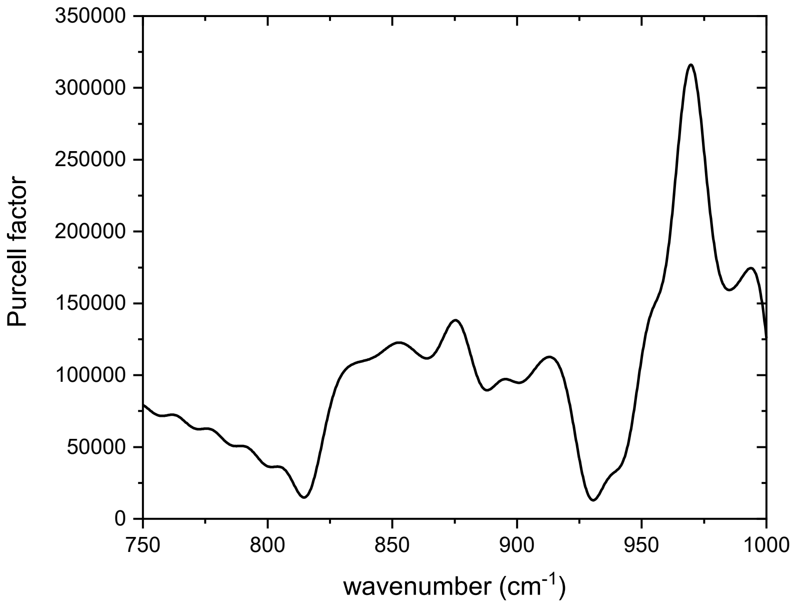

Figure S10. *Purcell factor dependence on the wavenumber for an emitter situated 10 nm above an α-MoO_3/4H-SiC system with a substrate with a carrier concentration of $N = 5 \times 10^{18}$ cm^{-3}.*

References

[1] N. T. Son, W. M. Chen, O. Kordina, and B. Monemar, "Electron effective masses in 4H SiC," *Appl Phys Lett*, vol. 66, pp. 1074–1076, 1995, https://doi.org/10.1063/1.113576.

[2] N. Son, P. Hai, W. Chen, C. Hallin, B. Monemar, and E. Janzén, "Hole effective masses in *4H-SiC*," *Phys Rev B*, vol. 61, no. 16, p. R10544, 2000, https://doi.org/10.1103/PhysRevB.61.R10544.

[3] M. Roschke and F. Schwierz, "Electron mobility models for 4H, 6H, and 3C SiC," *IEEE Trans Electron Devices*, vol. 48, no. 7, pp. 1442–1447, 2001, https://doi.org/10.1109/16.930664.

[4] D. W. Feldman, J. H. Parker, W. J. Choyke, and L. Patrick, "Phonon dispersion curves by raman scattering in SiC, polytypes 3C, 4H, 6H, 15R, and 21R," *Physical Review*, vol. 173, no. 3, pp. 787–793, 1968, https://doi.org/10.1103/PHYSREV.173.787.

[5] D. B. M. Klaassen, "A unified mobility model for device simulation—I. Model equations and concentration dependence," *Solid State Electron*, vol. 35, no. 7, pp. 953–959, 1992, https://doi.org/10.1016/0038-1101(92)90325-7.

[6] B. T. Spann *et al.*, "Photoinduced tunability of the reststrahlen band in *4H−SiC*," *Phys Rev B*, vol. 93, no. 8, p. 085205, 2016, https://doi.org/10.1103/PhysRevB.93.085205

[7] J. D. Caldwell *et al.*, "Low-loss, extreme subdiffraction photon confinement via silicon carbide localized surface phonon polariton resonators," *Nano Lett*, vol. 13, no. 8, pp. 3690–3697, 2013, https://doi.org/10.1021/nl401590g.

[8] Y. Chen *et al.*, "Spectral Tuning of Localized Surface Phonon Polariton Resonators for Low-Loss Mid-IR Applications," *ACS Photonics*, vol. 1, no. 8, pp. 718–724, 2014, https://doi.org/10.1021/ph500143u

[9] T. Wang, P. Li, B. Hauer, D. N. Chigrin, and T. Taubner, "Optical Properties of Single Infrared Resonant Circular Microcavities for Surface Phonon Polaritons," *Nano Lett*, vol. 13, no. 11, pp. 5051–5055, 2013, https://doi.org/10.1021/nl4020342.

[10] D. Navajas, J. M. Pérez-Escudero, M. E. Martínez-Hernández, J. Goicoechea, and I. Liberal, "Addressing the Impact of Surface Roughness on Epsilon-Near-Zero Silicon Carbide Substrates," *ACS Photonics*, vol. 10, no. 9, pp. 3105–3114, 2023, https://doi.org/10.1021/acsphotonics.3c00476.

[11] E. Janzén *et al.*, "Defects in SiC," *Physica B Condens Matter*, vol. 340–342, pp. 15–24, 2003, https://doi.org/10.1016/j.physb.2003.09.001.

[12] P. Ščajev, V. Gudelis, K. Jarašiūnas, and P. B. Klein, "Fast and slow carrier recombination transients in highly excited 4H– and 3C–SiC crystals at room temperature," *J Appl Phys*, vol. 108, no. 2, p. 023705, 2010, https://doi.org/10.1063/1.3459894.

[13] J. Li, P. Batoni, and R. Tsu, "Deposition of 4H-SiC on C-plane Sapphire with C_{60}," *ECS Trans*, vol. 25, no. 12, pp. 105–109, 2009, https://doi.org/10.1149/1.3238213.

[14] T.-T. Luong *et al.*, "2H-silicon carbide epitaxial growth on c-plane sapphire substrate using an AlN buffer layer and effects of surface pre-treatments," *Electronic Materials Letters*, vol. 11, no. 3, pp. 352–359, 2015, https://doi.org/10.1007/s13391-015-4208-9.

[15] M. C. Luo *et al.*, "Epitaxial growth and characterization of SiC on C-plane sapphire substrates by ammonia nitridation," *J Cryst Growth*, vol. 249, no. 1–2, pp. 1–8, 2003, https://doi.org/10.1016/S0022-0248(02)02019-5.

[16] J. Hwang, M. Kim, V. B. Shields, and M. G. Spencer, "CVD growth of SiC on sapphire substrate and graphene formation from the epitaxial SiC," *J Cryst Growth*, vol. 366, pp. 26–30, 2013, https://doi.org/10.1016/j.jcrysgro.2012.12.136.

[17] Z. Zheng *et al.*, "A mid-infrared biaxial hyperbolic van der Waals crystal," *Sci Adv*, vol. 5, no. 5, p. eaav8690, 2019, https://doi.org/10.1126/sciadv.aav8690.

[18] T. Zhan, X. Shi, Y. Dai, X. Liu, and J. Zi, "Transfer matrix method for optics in graphene layers," *Journal of Physics: Condensed Matter*, vol. 25, no. 21, p. 215301, 2013, https://doi.org/10.1088/0953-8984/25/21/215301.

[19] S. G. Menabde, J. T. Heiden, J. D. Cox, N. A. Mortensen, and M. S. Jang, "Image polaritons in van der Waals crystals," *Nanophotonics*, vol. 11, no. 11, pp. 2433–2452, 2022, https://doi.org/10.1515/nanoph-2021-0693

[20] A. Faraon, P. E. Barclay, C. Santori, K. M. C. Fu, and R. G. Beausoleil, "Resonant enhancement of the zero-phonon emission from a colour centre in a diamond cavity," *Nat Photonics*, vol. 5, no. 5, pp. 301–305, 2011, https://doi.org/10.1038/NPHOTON.2011.52.

[21] Ardavan Oskooi and Steven G. Johnson, "Electromagnetic Wave Source Conditions," in *Advances in FDTD Computational Electrodynamics: Photonics and Nanotechnology*, A. Taflove, A. Oskooi, and S. G. Johnson, Eds., Artech House, 2013, ch. 4, pp. 65–96.
